# Computer use at work is associated with self-reported depressive and anxiety disorder

**DOI:** 10.1186/s40557-016-0146-8

**Published:** 2016-10-13

**Authors:** Taeshik Kim, Mo-Yeol Kang, Min-sang Yoo, Dongwook Lee, Yun-Chul Hong

**Affiliations:** 1 Department of Preventive Medicine, Seoul National University, College of Medicine, 103 Daehangno, Jongno-gu Seoul, 110-799 Republic of Korea; 2 Institute of Environmental Medicine, Seoul National University Medical Research Center, 103 Daehangno, Jongno-gu Seoul, 110-799 Republic of Korea; 3 Seoul Gangseo Workers Health Center, Seoul, Republic of Korea; 4 Republic of Korea Navy Headquarters, Gyeryong, Republic of Korea

**Keywords:** Computer use, Depressive disorder, Anxiety disorder, KWCS, Working hours

## Abstract

**Background:**

With the development of technology, extensive use of computers in the workplace is prevalent and increases efficiency. However, computer users are facing new harmful working conditions with high workloads and longer hours. This study aimed to investigate the association between computer use at work and self-reported depressive and anxiety disorder (DAD) in a nationally representative sample of South Korean workers.

**Methods:**

This cross-sectional study was based on the third Korean Working Conditions Survey (2011), and 48,850 workers were analyzed. Information about computer use and DAD was obtained from a self-administered questionnaire. We investigated the relation between computer use at work and DAD using logistic regression.

**Results:**

The 12-month prevalence of DAD in computer-using workers was 1.46 %. After adjustment for socio-demographic factors, the odds ratio for DAD was higher in workers using computers more than 75 % of their workday (OR 1.69, 95 % CI 1.30−2.20) than in workers using computers less than 50 % of their shift. After stratifying by working hours, computer use for over 75 % of the work time was significantly associated with increased odds of DAD in 20–39, 41–50, 51–60, and over 60 working hours per week. After stratifying by occupation, education, and job status, computer use for more than 75 % of the work time was related with higher odds of DAD in sales and service workers, those with high school and college education, and those who were self-employed and employers.

**Conclusions:**

A high proportion of computer use at work may be associated with depressive and anxiety disorder. This finding suggests the necessity of a work guideline to help the workers suffering from high computer use at work.

**Electronic supplementary material:**

The online version of this article (doi:10.1186/s40557-016-0146-8) contains supplementary material, which is available to authorized users.

## Background

Depressive and anxiety disorders are common mental disorders contributing to major burdens of disease and are expected to result in work impairment and decreased quality of life. According to a recent report of the 2011 Epidemiological Survey of Mental Disorders among Korean Adults, the 1-year prevalence of depressive and anxiety disorder in South Korea was around 2 % and 6 % respectively [1, 2]. Contribution of the total disability-adjusted life years (DALYs) of depressive and anxiety disorder to a total burden of disease in South Korea were 2.66 % and 1.43 % in 2010 [3]. Moreover, depressive and anxiety disorders are suspected to be the leading cause of disability in high-income countries by 2030 [4].

With the development of information and communication technology, computer works have become common in advanced countries in a wide variety of workplaces ranging from clerical to sales. A recent report by the Korean government showed that 70.3 % of companies had at least one computer and that more than 80 % of the workers used computers more than once a week in 78.3 % of the companies [5]. Computer work has been demonstrated to be related to various adverse health effects with increased mental demands and workload [6–8]. These effects include work-related musculoskeletal disorders (WMSD), which are one of the most prevalent occupational diseases and also known as visual display terminal (VDT) syndrome [9]. Some reports have suggested that computer use at work is related to sleep disorder and subjective mental symptoms including depression and anxiety. In a study of Japanese administrative office workers, VDT use was significantly associated with neck and upper extremity pain as well as psychological distress [10, 11]. VDT workers in Italy had significantly higher total stress scores compared to office workers who were not assigned to VDT jobs. However, the research on the effect of computer use at work is still inconclusive, especially with regard to psychological symptoms and mental disorders [12]. Compared to the musculoskeletal disorders, few studies have investigated associations between computer use at work and mental disorders. Because the previous studies about computer work mostly focused on specific groups of workers such as administrative office workers and there are no known studies on the general working population, more research is needed to investigate the association between computer work and mental disorders in general working population.

Computer workers in the general working population are diverse in terms of socio-economic status and work type. For instance, call center workers are exposed to different working conditions compared to senior managers. Low socio-economic status was generally related to a high prevalence of common mental disorders in previous studies [13–15]. Occupational group and employment status were also important regarding factors affecting mental disorders [16, 17]. Therefore, it is important to consider the modifying effect of occupational group, education, and job status on the association between computer work and mental disorders in the general population. Korean workers work the second longest hours among the Organization for Economic Cooperation and Development (OECD) countries and long working hours were associated with an increased injury rate, behavioral problems and various adverse health effects such as depressive states, anxiety, sleep disorders, and coronary heart disease [18, 19]. Therefore, working hours for Korean workers need to be considered as a modifying factor in the association between computer works and mental disorders.

The aim of this study is to investigate the association between computer work time and self-reported depressive and anxiety disorder in a nationally representative sample of South Korean workers as well as to examine the modifying effects of working hours, occupational group, education, and job status on the associations between computer work time and depressive and anxiety disorder.

## Methods

### Participants

We investigated data collected from the Third Korean Working Conditions Survey 2011 (KWCS), a nationally representative cross-sectional survey. KWCS, benchmarking the European Working Conditions Survey (EWCS) and the UK Labour Force Survey, has been conducted by the Korean Occupational Safety and Health Research Institute since 2006. This study was conducted using a stratified and clustered sampling from the National Population and Housing Census. The survey population consisted of individuals 15 years of age and older who had actively worked more than an hour in the past week, with the exception of prisoners, island, and special facilities residents. Of the total 50,032 participants, 48,850 workers who worked more than 20 h per week were included in this study.

### Measurements

Computer use: The computer use variable was assessed by the question, “Working with computers: PCs, network, mainframe” with 7 response options: never, almost never, around 1/4 of the time, around half of the time, around 3/4 of the time, almost the time, and all of the time. Computer use time proportion was classified into 3 categories (<1/2 of the time, 1/2 ~ 3/4 of the time, and >3/4 of the time).

Working hours: Working hours were evaluated by the answer to the open question “How many hours do you usually work per week in your main paid job?” Working hours were categorized into the following 5 groups: 20–39 h, 40 h, 41–50 h, 51–60 h, and above 60 h per week. We used 40 h per week as the reference group.

Self-reported depressive and anxiety disorder: Self-reported depressive and anxiety disorder was defined as a response of “yes” to the question “Over the last 12 months, did you suffer from any of the following health problems: Depression and anxiety disorder?”

Socio-demographic characteristics: Socio-demographic factors were analyzed as covariates including sex, age, education level, and occupational group. Education level was classified into 4 groups: middle school or lower, high school, college, and university graduate or above. The 9 categories of occupational groups (professionals, senior managers, clerical workers, sales workers, service workers, highly skilled worker, semi-skilled workers, unskilled workers, and agricultural/fishery workers) were recategorized into 4 groups: professionals and senior managers, clerical workers, sales and service workers, and manual workers. Job status was divided into 3 categories of fulltime employees, part-time employees, and a third category including self-employed, employers, and others. Smoking status was classified using the dichotomous variable: “current non-smoker” and “current smoker”. Problematic alcohol drinking was categorized as drinking more than 6 glasses per week for males and drinking more than 4 glasses of alcohol for females.

### Statistical analysis

Socio-demographic factors were described using mean and proportion. The frequency of computer use at work for more than 1/4 of working hours was described. Prevalence of self-reported depressive and anxiety disorder (DAD) was analyzed by *χ* ^2^ test. The odds ratio of DAD by computer work time was assessed by multiple logistic regression adjusting for socio-demographic factors. Model 1 was a regression model adjusted for sex and age. In Model 2, socio-demographic factors including sex, age, education, occupational group, job status, working hours, problem drinking, and current smoking were adjusted. Combined effects of computer use during work time and total working hours per week was calculated using logistic regression after adjusting socio-demographic factors in Model 2. We stratified each computer use proportion by the 5 categories of working hours for analysis. The association between DAD and computer use by occupational group, education, and job status was investigated with logistic regression adjusted for socio-demographic variables. All analyses were performed using SAS 9.3 statistical software for Windows (SAS Inc., Cary, NC, USA).

## Results

Socio-demographic characteristics for the study participants are shown in Table 1. Mean age was 45.8 years old (SD = 13.1) and 57.8 % were men. In regard to occupational classification, manual workers accounted for the largest number (37.1 %), followed by sales and service (32.4 %) as the second largest occupational group. The majority of the participants (40.9 %) completed a high school education. Of the 48,850 workers, 22.3 % reported having an alcohol drinking problem and 30.0 % were currently smoking. The overall prevalence of self-rated DAD was 1.46 %. The DAD prevalence was higher in females, workers over 50 years old, sales and service workers, and those with less than a high school education. Workers with long working hours more than 61 h per week had high DAD prevalence.

**Table 1:** Socio-demographic characteristics of study participants by computer user and self-reported depressive and anxiety disorder (DAD)

Characteristic	Total	Computer worker^a^		DAD prevalence	
	N (%)	N (%)	*P*-value	%	*P*-value
Sex					
Male	28253 (57.8)	10671 (37.8)	<0.001	1.19	<0.001
Female	20597 (42.2)	6848 (33.3)		1.83	
Age					
15–29	5091 (10.4)	2496 (49.0)	<0.001	1.12	<0.001
30–39	11471 (23.5)	6117 (53.3)		1.27	
40–49	14147 (29.0)	5833 (41.2)		1.43	
50-	18141 (37.1)	3073 (16.9)		1.69	
Occupational group					
Professionals + Senior managers	7431 (15.2)	5317 (71.6)	<0.001	1.28	0.055
Clerical	6691 (13.7)	5868 (87.7)		1.21	
Sales + service	15817 (32.4)	4280 (27.1)		1.62	
Manual worker	18910 (38.7)	2054 (10.9)		1.48	
Education					
Middle School or lower	9099 (18.6)	361 (4.0)	<0.001	1.92	<0.001
High School	19875 (40.9)	4447 (22.4)		1.51	
College	7617 (15.6)	3819 (50.1)		1.13	
University graduate or above	12259 (25.1)	8892 (72.5)		1.22	
Job status					
Self-employed, employer, and others	19886 (40.7)	4417 (22.2)	<0.001	1.69	0.010
Full-time employee	27134 (55.6)	12848 (47.4)		1.25	
Part-time employee	1830 (3.8)	254 (13.9)		1.97	
Working hours					
20–39 h	4763 (9.8)	719 (15.1)	<0.001	2.16	<0.001
40 h	10767 (22.0)	6221 (57.8)		1.07	
41–50 h	13448 (27.5)	5786 (43.0)		1.44	
51–60 h	11145 (22.8)	3107 (27.9)		1.15	
61 h-	8727 (17.9)	1686 (19.3)		1.97	
Current Smoking status					
Non-smoking	34216 (70.0)	121814 (35.6)	0.074	1.57	0.002
Smoking	14634 (30.0)	5335 (36.5)		1.20	
Problem drinking					
Yes	10920 (22.3)	4490 (41.1)	<0.001	1.47	0.821
No	37930 (77.7)	13029 (34.4)		1.43	
Total	48850 (100.0)	17519 (35.9)		1.46	

^a^Computer worker is determined as using a computer for more than 1/4 of total work time following the definition of EWCS

Table 2 illustrates the association between computer work time and DAD. After adjustment for sex and age (Model 1), use of a computer for >3/4 of work time had a significant association with DAD compared to those who used a computer <1/2 of their work time (Odds ratio 1.34, 95 % CI 1.08–1.68). After adjusting for sex, age, education, occupational group, job status, working hours, problem drinking, and current smoking (Model 2), use of a computer >3/4 of the work time was significantly associated with DAD (Odds ratio 1.69, 95 % CI 1.30–2.20).

**Table 2:** Association of computer use time and self-reported depressive and anxiety disorder (DAD)

Computer use	N (%)	DAD Prevalence (%)	Model 1	Model 2
			OR (95 % CI)	OR (95 % CI)
<1/2	36168 (74.0)	1.46	1	1
1/2–3/4	6262 (12.8)	1.29	1.04 (0.82–1.33)	1.27 (0.97–1.65)
>3/4	6420 (13.1)	1.62	1.34 (1.08–1.68)	1.69 (1.30–2.20)
p for trend			0.015	<0.001

Model 1: adjusted for sex and age

Model 2: adjusted for sex, age, education, occupational group, job status, working hours, problem drinking, and current smoking

The combined relationship of the proportion of computer use during work time and the total working hours with DAD was described in Table 3. After adjusting for socio-economic factors, computer use >3/4 of the time of 41–50 work hours, 51–60 work hours, and more than 60 work hours per week was significantly related to increased odds of DAD compared to those who worked 40 h a week. Computer use less than 1/2 of more than 60 working hours per week was also associated with significantly higher odds of DAD. For short work hours, > 3/4 computer use of the work time had a higher odds ratio (OR) for DAD. After being stratified by working hours (Table 3), the relationship between computer use and DAD had a significant dose-response relationship as computer use proportion increased (p for trend = 0.003, 0.020, 0.048). Interaction between computer use and working hours was significant (p value = 0.0078).

**Table 3:** Adjusted odds ratios (95 % CI)^a^ trend of DAD for combined effect and by interactions of computer use and working hours

Working hours					
Computer use	20–39 h	40 h	41–50 h	51–60 h	61 h -
combined effect					
<1/2	1.3 (0.93–1.81)	1	1.02 (0.76–1.37)	0.73 (0.53–1.00)	1.39 (1.03–1.87)
1/2–3/4	1.98 (0.84–4.67)	0.74 (0.43–1.27)	1.51 (0.98–2.31)	1.51 (0.90–2.53)	0.92 (0.4–2.15)
>3/4	2.48 (1.12–5.53)	0.86 (0.52–1.40)	1.88 (1.24–2.86)	2.12 (1.26–3.57)	2.74 (1.55–4.86)
by interactions					
<1/2	1	1	1	1	1
1/2–3/4	1.85 (0.74–4.61)	0.74 (0.41–1.33)	1.49 (0.96–2.3)	1.63 (0.92–2.89)	0.71 (0.31–1.66)
>3/4	2.64 (1.07–6.52)	0.89 (0.51–1.57)	1.91 (1.23–2.97)	2 (1.08–3.68)	2.13 (1.18–3.84)
p for trend	0.023	0.636	0.003	0.020	0.048

^a^Adjusted for sex, age, education, occupational group, job status, problem drinking, and current smoking (Model 2)

In order to investigate the association between computer use and DAD in participants from different occupational groups, education, and job status, stratified analyses were applied as seen in Additional file 1: Tables S1. In sales and service workers, computer use for 1/2 ~ 3/4 of the work time (OR 1.66, 95 % CI 1.11–2.48) and for >3/4 of the work time (OR 2.07, 95 % CI 1.35–3.17) had higher OR for DAD compared to computer use for <1/2 of the work time. In other occupational group, computer use time was not significantly related with DAD. The combined effect of computer use and occupational group on DAD, shown in Additional file 2: Table S2, produced no statistically significant relationship. In regard to education, high school and college education had a significantly high OR in workers who used computers for >3/4 of the work time (Additional file 1: Table S1). The university graduate or above group had a marginal association with DAD for computer use for >3/4 of work time, and the combined effect of computer use and education on DAD is displayed in Additional file 2: Table S2. When examining job status, responses of self-employed, employer, and the others had a statistically significant association with DAD only for those using computers for >3/4 of their work time (OR 2.22, 95 % CI 1.46–3.38; Additional file 1: Table S1). In full-time employees, computer use for >3/4 of the work time was related to DAD (OR 1.46, 95 % CI 1.04–2.05). The combined effect of computer use and education on DAD is exhibited in Additional file 2: Table S2.

## Discussion

The aim of this study was to investigate the association between computer use at work and self-reported depressive and anxiety disorder in a nationally representative sample of Korean workers. The results showed that participants using computers for more than 3/4 of the work time had a higher odds ratio of DAD after adjusting for socio-demographic factors. From combined association of computer use proportion and the total working hours, this study revealed that computer use for >3/4 of the work time was associated with DAD for both short and long working hours. Sales and service workers as well as those with high school and college education, with a high proportion of computer use had a significantly higher odds ratio of DAD with an increasing trend. For job status, the third category of self-employed, employers, and the others and the category of full-time employees were significantly related to a higher odds ratio of DAD with a dose-response relationship.

This result is consistent with other previous studies. Ye et al. has shown that over 5 h of computer work is associated with high scores on the 12-item General Health Questionnaire, a screening instrument for common mental disorders and psychiatric well-being. Nakazawa et al. demonstrated that adverse mental symptom scores in computer workers were significantly related with using VDT for a long duration compared to using VDT for a short duration [10]. Moreover, several studies about the relationship between daily computer use and mental health among students and young adults have indicated that high use of computers or internet addiction increased pathologic symptoms such as depression, sleep disturbances, and insomnia [20–22].

Several hypotheses have been proposed. Firstly, computer work could be a source of job stress which is highly mentally demanding with requiring more attention and cognitive concentration [7, 23]. Although computers made the tasks efficiently processed, work intensification was also simultaneously developed. Work intensification is defined as an increase in the demands, pace, or qualitative requirements of the tasks and may be related to a high level of job strain and distress. In the highly skilled workers, creative and complexed tasks are demanded by using computers. In the low skilled workers, almost computers works are highly controlled and repetitive. These tasks are monotonous but still requiring constant attention such as accounting in sales department and handling customer service with information (e.g., customers’ call center). In both area, computer works are mentally demanding and therefore, job stress can be high and cognitive problems such as concentration, memory, and decision-making could be affected by increased computer job demands [23]. Further, these job stress can be aggravated with work intensification. With the technology developed, computer use may intensify the pace of work with computer monitoring system and multitasking [7, 24, 25]. Secondly, computer works are usually highly sedentary. Recently, systematic reviews investigated that sedentary works including computer work were related with depression and anxiety [26–28]. Decreased physical activity could be one of the mechanism of sedentary behaviors related depression and anxiety. Thirdly, social withdrawal hypothesis suggested that computer work could remove individuals from social interactions and increase risk for depression [7, 24]. Fourthly, technology-related stress which is caused by frustrating experiences with hardware or software problems can increase stress and anxiety in computer workers with high job demands. Lastly, sleep problems and musculoskeletal disorders related with computer work could affect the pathophysiology of the development of depression and anxiety [29, 30].

We found that computer use for more than 3/4 of total work time is associated with a more than 2-fold odds ratio of DAD among the workers with both short and long working hours. There was a dose-response relationship between computer use and DAD after stratification by working hours. Many previous studies have revealed that long working hours are related to depression and anxiety disorders [31, 32]. In addition to long working hours, high computer use may have additional psychologically adverse health effects through increased job stress, work intensification and the pervasive use of computers in the home.. With work intensification, the pervasive use of the computer and work extension can blur the boundaries between work and private life, which can further increase demands and responsibilities and shorten time for private life and leisure.

Previous studies have found that short working hours were related to a higher prevalence of depression, possibly due to other chronic illnesses. The U-shaped relationship between working hours and health outcomes has been investigated in previous studies [33], especially in regard to cardiovascular diseases, and could be explained by the healthy worker effect [34]. Individuals who work less than 40 h per week may be precarious workers such as part-time workers or temporary workers. Recently, many studies have revealed that precarious work is related to deteriorated mental health and that it is bi-directionally associated with mental disorders [35, 36].

In the general working population, it is important to identify the groups vulnerable to DAD. In order to evaluate the modifying effect of job type on DAD, we stratified the occupational groups into 4 categories. Clerical and professional working groups were the 1 doing the most computer work, accounting for 64 % of the total computer workers, while substantial computer work was also performed by service, sales, and manual workers, accounting for 36 %. Sales and service workers with high computer use were linked to DAD, while clerical and professional workers were not. As previously mentioned, sales and service workers in computer work are faced with high job demands and low job control [7]. The greater amount of computer work may increase the monotony and tedium of their job and exacerbate job stress with poor psychosocial working condition [24, 37]. In addition, emotionally stressful tasks dealing with customers and complaints such as call centers can have adverse effects on mental health with low job control and high demands [38]. Low support from supervisor and co-workers can increase the risk for DAD with electronic operating and monitoring systems [39]. In manual workers, the relationship between computer use and DAD was not statistically significant probably because of the small number of computer users. However, high school and college education were related to an increased odds ratio of DAD. Previous studies revealed that low socioeconomic status, including low education level, was generally connected to common mental disorders and persistent morbidity [13–15]. Additionally, because more monotonous and repetitive tasks combined with high job demands and low job control tend to be allocated more in low-educated groups than in highly educated groups, they could be affected by high job strain, a known risk factor for depression and anxiety. Job insecurity and low opportunity of career advancement in low education level may also increase the risk for DAD [40].

Self-employed, employer, and others using computers for >3/4 of the work time had a significantly higher odds ratio of DAD with a dose-response relationship between computer use and DAD. The significantly elevated risk of DAD among the self-employed or employers may be partly due to work engagement or workaholism without social contact [41]. Besides, 80.7 % of these workers excluding employer with employee and entrepreneurs were self-employed, unpaid family workers and other workers which can be categorized into informal employment. They are away from job security, social support, and workplace regulations and are exposed to the risk factors of DAD [42]. Full-time employees using computers for >3/4 of the work time also had a higher odds ratio of DAD with a dose-response relationship. However, in part-time employees we could not observe the relationship between computer use and DAD due to small numbers of high computer users.

Several limitations of this study should be mentioned. First, DAD was detected by a single question on a binary scale about the experience of the disorders in the last 12 months. Therefore, reliability was not assured. A validated instrument or professional diagnosis could strengthen the reliability of the definition of the mental disorders. Second, although we used large representative worker data, there was a limitation in the relatively small number reporting DAD. The prevalence of DAD (1.46 %) was lower than in previous Korean mental health surveys (2 % and 6 %). Third, as KWCS had a cross-sectional design, the association between computer use and DAD could be bi-directional and the definite causality of computer work to DAD could not be investigated. Depressive workers might perform more slowly or work shorter hours than the euthymic workers. Fourth, KWCS used a categorical question about the proportion of computer use during work time. Therefore, the effect of the actual duration of computer use could not be investigated. Lastly, although KWCS was designed using stratified clustered sampling, we could not analyze KWCS with survey weight approaches due to limited data availability.

Based on our results, computer use at work was associated with DAD in South Korea. We need to recognize computer use as risk factor of depressive and anxiety disorders. Computer work more than 3/4 of the work time could be related with mental health problems and the association could be higher in worker with long working hours more than 50 h. Especially, workers with low education, low skilled, and self-employed workers are highly associated with DAD and could be susceptible group to protect from computer use and other poor working conditions. Management of psychosocial working conditions includes long working hours, high demands, low control, and low social support have to be considered in these susceptible groups. Organizational support including job security and social interaction could be a start point of improving working conditions and the effort to reduce job demands and enhance the job control are needed. Personal effort are also important in balancing work and private spheres which are blurred by the computer work systems. Since in real situations these suggestions for improvement of working conditions are hardly achieved by only the effort of enterprises and individuals, the organizational support of government are needed at this point which includes policies and guidelines of improvement of working condition and promotion of workplace health for computer work.

## Conclusions

Longer hours of computer work was associated with an increased risk of DAD in South Korean workers. To our knowledge, this study is the first report demonstrating the association between computer use at work and DAD in a representative sample from a national population census. In the sales and service workers, those with a high school or college education, and the self-employed and employers, the association between computer use and DAD was prominent. This finding may be helpful in developing workplace health promotion guidelines for workers suffering from high computer use at work.

## Additional files

Additional file 1: Table S1. Adjusted OR* of DAD by interaction of computer use during work time and occupational group, education, and job status. (DOC 59 kb)
Additional file 2: Table S2. Adjusted OR* of DAD considering the combined effect of computer use and occupational group, education, and job status. (DOC 61 kb)

## Abbreviations

DAD: Depressive and anxiety disorder
EWCS: European Working Conditions Survey
KWCS: Korean Working Conditions Survey
VDT: Visual display terminal
WMSD: Work-related musculoskeletal disorders
