# Supplementary material for: Computer use at work is associated with self-reported depressive and anxiety disorder
Source: Ann Occup Environ Med. 2016 Oct 13;28:57. doi: 10.1186/s40557-016-0146-8 (PMC5062816; doi:10.1186/s40557-016-0146-8)
Supplement: Additional file 1: Table S1. — Adjusted OR* of DAD by interaction of computer use during work time and occupational group, education, and job status. (DOC 59 kb) [file 40557_2016_146_MOESM1_ESM.doc]

Table S 1. Adjusted OR* of DAD by interaction of computer use during work time and occupational group, education, and job status

|  | Occupational group | | | | | | | | | | |  | Education | | | | | | | | | | |  | Job status | | | | | | | |
| --- | --- | --- | --- | --- | --- | --- | --- | --- | --- | --- | --- | --- | --- | --- | --- | --- | --- | --- | --- | --- | --- | --- | --- | --- | --- | --- | --- | --- | --- | --- | --- | --- |
| Computer  Use | Manual worker | |  | Sales+Service workers | |  | Clerical workers | |  | Professionals+ Senior managers | |  | <High school | |  | High school | |  | College | |  | University, Graduate school | |  | Self-employed, employer, and others | |  | Full-time employee | |  | Part-time employee | |
|  | N | OR  (95% CI) |  | N | OR  (95% CI) |  | N | OR  (95% CI) |  | N | OR  (95% CI) |  | N | OR  (95% CI) |  | N | OR  (95% CI) |  | N | OR  (95% CI) |  | N | OR  (95% CI) |  | N | OR  (95% CI) |  | N | OR  (95% CI) |  | N | OR  (95% CI) |
|  |  |  |  |  |  |  |  |  |  |  |  |  |  |  |  |  |  |  |  |  |  |  |  |  |  |  |  |  |  |  |  |  |
| <1/2 | 17772 | 1 |  | 13208 | 1 |  | 1367 | 1 |  | 3821 | 1 |  | 8900 | 1 |  | 16992 | 1 |  | 4897 | 1 |  | 5379 | 1 |  | 17233 | 1 |  | 17295 | 1 |  | 1640 | 1 |
| 1/2-3/4 | 704 | 1.36  (0.71-2.61) |  | 1520 | **1.66**  **(1.11-2.48)** |  | 2153 | 0.65  (0.33-1.27) |  | 1885 | 1.16  (0.69-1.93) |  | 121 | 0.52  (0.07-3.82) |  | 1543 | 1.04  (0.66-1.65) |  | 1405 | 1.57  (0.88-2.79) |  | 3193 | 1.24  (0.81-1.89) |  | 1563 | 1.16  (0.73-1.83) |  | 4596 | 1.2  (0.85-1.69) |  | 103 | 2.93  (0.89-9.71) |
|  |  |  |  |  |  |  |  |  |  |  |  |  |  |  |  |  |  |  |  |  |  |  |  |  |  |  |  |  |  |  |  |  |
| >3/4 | 434 | 0.98  (0.36-2.71) |  | 1090 | **2.07**  **(1.35-3.17)** |  | 3171 | 1.24  (0.7-2.18) |  | 1725 | 1.49  (0.91-2.44) |  | 78 | 0.85  (0.11-6.33) |  | 1340 | **1.75**  **(1.13-2.70)** |  | 1315 | **2.25**  **(1.25-4.05)** |  | 3687 | 1.43  (0.95-2.16) |  | 1090 | **2.22**  **(1.46-3.38)** |  | 5243 | **1.46**  **(1.04-2.05)** |  | 87 | 1.13  (0.21-6.02) |
| p for trend |  | 0.637 |  |  | <0.001 |  |  | 0.228 |  |  | 0.120 |  |  | 0.637 |  |  | 0.024 |  |  | 0.006 |  |  | 0.087 |  |  | <0.001 |  |  | 0.028 |  |  | 0.439 |

* Adjusted for sex, age, education, occupational group, job status, working hours, problem drinking and current smoking
