# Supplementary material for: Computer use at work is associated with self-reported depressive and anxiety disorder
Source: Ann Occup Environ Med. 2016 Oct 13;28:57. doi: 10.1186/s40557-016-0146-8 (PMC5062816; doi:10.1186/s40557-016-0146-8)
Supplement: Additional file 2: Table S2. — Adjusted OR* of DAD considering the combined effect of computer use and occupational group, education, and job status. (DOC 61 kb) [file 40557_2016_146_MOESM2_ESM.doc]

Table S 1. Adjusted OR* of DAD considering the combined effect of computer use and occupational group, education, and job status

|  | Occupational group | | | | | | | | | | |  | Education | | | | | | | | | | |  | Job status | | | | | | | |
| --- | --- | --- | --- | --- | --- | --- | --- | --- | --- | --- | --- | --- | --- | --- | --- | --- | --- | --- | --- | --- | --- | --- | --- | --- | --- | --- | --- | --- | --- | --- | --- | --- |
| Computer  Use | Manual worker | |  | Sales+Service workers | |  | Clerical workers | |  | Professionals+ Senior managers | |  | <High school | |  | High school | |  | College | |  | University, Graduate school | |  | Self-employed, employer, and others | |  | Full-time employee | |  | Part-time employee | |
|  | N | OR  (95% CI) |  | N | OR  (95% CI) |  | N | OR  (95% CI) |  | N | OR  (95% CI) |  | N | OR  (95% CI) |  | N | OR  (95% CI) |  | N | OR  (95% CI) |  | N | OR  (95% CI) |  | N | OR  (95% CI) |  | N | OR  (95% CI) |  | N | OR  (95% CI) |
|  |  |  |  |  |  |  |  |  |  |  |  |  |  |  |  |  |  |  |  |  |  |  |  |  |  |  |  |  |  |  |  |  |
| <1/2 | 17772 | 0.77  (0.47-1.26) |  | 13208 | 0.73  (0.44-1.2) |  | 1367 | 1 |  | 3821 | 0.74  (0.43-1.28) |  | 8900 | 1.17  (0.81-1.68) |  | 16992 | 1.12  (0.82-1.54) |  | 4897 | 0.81  (0.54-1.2) |  | 5379 | 1 |  | 17233 | 1.02  (0.83-1.25) |  | 17295 | 1 |  | 1640 | 1.06  (0.69-1.61) |
| 1/2-3/4 | 704 | 0.99  (0.46-2.16) |  | 1520 | 1.24  (0.69-2.23) |  | 2153 | 0.61  (0.32-1.18) |  | 1885 | 0.91  (0.5-1.68) |  | 121 | 0.62  (0.08-4.51) |  | 1543 | 1.26  (0.76-2.07) |  | 1405 | 1.2  (0.71-2.05) |  | 3193 | 1.35  (0.89-2.03) |  | 1563 | 1.15  (0.73-1.8) |  | 4596 | 1.24  (0.89-1.73) |  | 103 | 2.79  (0.99-7.8) |
|  |  |  |  |  |  |  |  |  |  |  |  |  |  |  |  |  |  |  |  |  |  |  |  |  |  |  |  |  |  |  |  |  |
| >3/4 | 434 | 0.66  (0.22-1.96) |  | 1090 | 1.56  (0.86-2.84) |  | 3171 | 1.1  (0.64-1.9) |  | 1725 | 1.23  (0.68-2.23) |  | 78 | 0.95  (0.13-6.96) |  | 1340 | **2.11**  **(1.35-3.31)** |  | 1315 | **1.69**  **(1.02-2.8)** |  | 3687 | **1.53**  **(1.03-2.27)** |  | 1090 | **2.21**  **(1.47-3.31)** |  | 5243 | **1.50**  **(1.08-2.07)** |  | 87 | 1.63  (0.39-6.75) |
|  |  |  |  |  |  |  |  |  |  |  |  |  |  |  |  |  |  |  |  |  |  |  |  |  |  |  |  |  |  |  |  |  |

* Adjusted for sex, age, education, occupational group, job status, working hours, problem drinking and current smoking
